# Exploring the Franck–Condon region of a photoexcited charge transfer complex in solution to interpret femtosecond stimulated Raman spectroscopy: excited state electronic structure methods to unveil non-radiative pathways†

**DOI:** 10.1039/d1sc01238j

**Published:** 2021-04-28

**Authors:** Federico Coppola, Paola Cimino, Umberto Raucci, Maria Gabriella Chiariello, Alessio Petrone, Nadia Rega

**Affiliations:** Department of Chemical Sciences, University of Napoli Federico II, Complesso Universitario di M.S. Angelo via Cintia Napoli 80126 Italy alessio.petrone@unina.it nadia.rega@unina.it; Department of Pharmaceutical Sciences, University of Salerno Salerno 84084 Italy; Centro Interdipartimentale di Ricerca sui Biomateriali (CRIB) Piazzale Tecchio Napoli I-80125 Italy

## Abstract

We present electronic structure methods to unveil the non-radiative pathways of photoinduced charge transfer (CT) reactions that play a main role in photophysics and light harvesting technologies. A prototypical π-stacked molecular complex consisting of an electron donor (1-chloronaphthalene, 1ClN) and an electron acceptor (tetracyanoethylene, TCNE) was investigated in dichloromethane solution for this purpose. The characterization of TCNE:π:1ClN in both its equilibrium ground and photoinduced low-lying CT electronic states was performed by using a reliable and accurate theoretical–computational methodology exploiting *ab initio* molecular dynamics simulations. The structural and vibrational time evolution of key vibrational modes is found to be in excellent agreement with femtosecond stimulated Raman spectroscopy experiments [R. A. Mathies *et al.*, *J. Phys. Chem. A*, 2018, **122**, 14, 3594], unveiling a correlation between vibrational fingerprints and electronic properties. The evaluation of nonadiabatic coupling matrix elements along generalized normal modes has made possible the interpretation on the molecular scale of the activation of nonradiative relaxation pathways towards the ground electronic state. In particular, two low frequency vibrational modes such as the out of plane bending and dimer breathing and the TCNE central C=C stretching play a prominent role in relaxation phenomena from the electronic CT state to the ground state one.

## Abstract 2

We present electronic structure methods to unveil the non-radiative pathways of photoinduced charge transfer (CT) reactions that play a main role in photophysics and light harvesting technologies.

## Graphical abstract

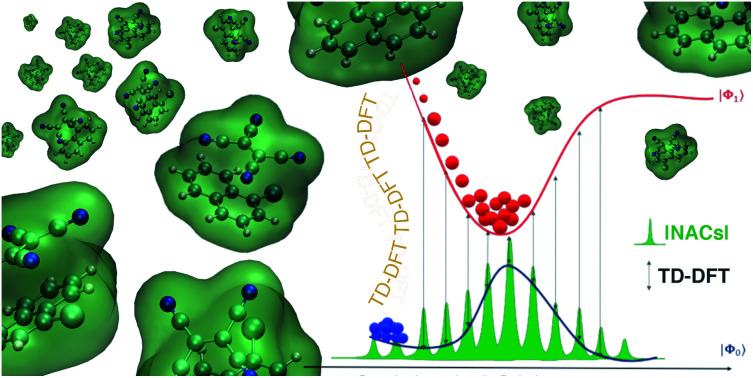

## 1 Introduction

The charge transfer (CT) process is a ubiquitous phenomenon of paramount importance in chemistry,^1,2^ biology^3,4^ and materials science.^5,6^ As a matter of fact, several studies on sustainable charge transfer systems employing organic molecules are emerging to increase their potential light harvesting capabilities to replace costly rare-earth metals and/or toxic materials, trying to understand how they can better generate excitons and other charge separated species.^7^ Electronic charge separation can be photoinduced at the interface between two materials^8,9^ in conjugated polymers,^10,11^ intra-^12–15^ or intermolecularly^16,17^ and in metal–ligand coordination complexes.^18–20^ A particular class of molecular compounds undergoing intermolecular CT is that of non-covalent π-stacked CT complexes, which are intensely colored molecular systems showing a weak interaction of a π-electron donor (D) and π-electron acceptor (A). Weak D–A complexes have attracted interest since the discovery of the conductive metal-like properties of the tetrathiafulvalene–tetracyanoquinodimethane complex in the early seventies^21–25^ and recently they have regained popularity due to their versatility in a wide range of fields such as organic opto-electronics,^26^ solar energy conversion^27–30^ and non-linear spectroscopy.^31–33^

According to Mulliken D–π–A complex theory,^34,35^ a CT complex is composed of two independent subunits forming a non-covalent dimer with an acid–base Lewis character, and thus a partial donation of electronic density stabilizes the dimer formation giving rise to mutual Coulomb interactions. When in solution, the complexation leads to a small charge transfer which strongly increases upon photoexcitation generating a CT state (D^*ρ*+^/A^*ρ*−^), whose electronic density rearranges faster compared to the nuclear motion in a very different manner with respect to the ground state one. The sudden electronic density reorganization induces relevant forces and structural changes, which can be monitored by ultrafast laser pulses in the fs regime, capable of probing the ultrafast nuclear reorganization in the so-called Franck–Condon region.^36–39^

As a matter of fact, steady-state vibrational spectroscopy and time-dependent variants pave the way to the investigation of fast transient dynamic photophysical and photochemical processes in real time. The correct band assignment of an experimental spectrum and the role played by specific vibrational modes that rule the relaxation dynamics in the excited state are not trivial tasks and several factors (anharmonicity, couplings, and environmental effects) must be taken into account.^40–45^ It is worth noticing that the electronic spectra of CT adducts show new absorption bands, absent in each monomer, that usually correspond to transitions from the donor–acceptor frontier orbitals. In particular, non-covalent molecular systems, such CT bands, are typically observed in well-planar aromatic molecules, where their interplanar distance and relative orientations play a key role in the CT event modulation and non-radiative decay pathways.^46,47^ On the other hand, as previously mentioned, time-resolved vibrational experiments can help monitor the interplay between electrons and nuclei in this non-equilibrium regime, where optical and vibrational properties constantly evolve in real time on femtosecond scales. An *ad hoc* protocol to finely interpret the output of time-resolved spectroscopic studies is still missing and no unique rigorous full quantum approach can be invoked to deal with the challenging time evolution of non-adiabatic quantum states. For these reasons, our efforts in this work are aimed at unraveling the excited state vibrational dynamics of the tetracyanoethylene:π:1-chloronaphthalene (TCNE:π:1ClN) dimer in dichloromethane DCM solution recently characterized by Mathies and coworkers by Femtosecond Stimulated Raman Spectroscopy (FSRS).^16^ TCNE is a typical electron acceptor molecule in non-covalent CT complexes and the photochemical phenomena underlying its non-radiative relaxation have recently been studied *via* non-adiabatic molecular dynamics.^47^

FSRS is an ultrafast vibrational spectroscopic technique which allows access to vibrational structural dynamics with a high temporal and spectral resolution (up to 50 fs and 10 cm^−1^, respectively),^36,48,49^ as well as the direct observation of anharmonic couplings in the time domain.^50^ These incredible features paved the way for the study of reactive events in the time domain in small molecules up to complex biological systems.^16,17,51–53^ In this work, we aim to face these challenges, proposing to apply an innovative theoretical protocol to directly observe the vibrational couplings and/or to reveal the frequencies underlying a signal oscillating over time. In this regard, we combined both non-equilibrium generalized normal modes and continuous wavelet transform to transient photoinduced phenomena affecting the CT in the Franck–Condon region. The ability of the method is shown for the first time to catch the instantaneous time-frequency correlations, making possible discerning (i) whether and what modes are coupled and (ii) when a specific mode is activated after the deactivation of other modes and provides a molecular picture to modern time resolved and non-linear spectroscopic experiments. This approach has been successfully used in recent years to analyse accurately equilibrium^54^ and transient phenomena such as time-resolved fluorescence,^55^ reactivity in excited states,^56,57^ polaron pair formation in excited semiconducting organic polymers^58^ and photorelaxation in solution.^59^ Given the requirement of dealing with electronic excited states and Franck–Condon relaxation, a detailed interpretation of such non-equilibrium phenomena at the molecular level can be only provided by *ab initio* electronic structure methods, which explicitly describes the electronic density and its response to an external perturbing field. As an additional challenge, the required theory has to simultaneously describe with high accuracy the weak dispersion forces between the subunits, during non-equilibrium processes, and the relaxation dynamics involving multiple and sometimes coupled electronic states.^60–66^ From the modeling perspective, π-stacked CT systems also require a very good description of both non-covalent interactions and spatially delocalized excitations (*i.e.*, valence-Rydberg, doubly excited, ππ*). In this context, we propose to study the model system *via ab initio* molecular dynamics simulations (AIMD)^67–71^ for the ground and the excited state in which energies and forces are computed within methods rooted in Density Functional Theory (DFT) and its time-dependent extension (TD-DFT) for the excited state properties. DFT and TD-DFT due to their relatively lower computational cost compared with *ab initio* post Hartree–Fock multiconfigurational wavefunction methods and competitive accuracy are today the most widely used electronic structure methods that can aid the study of problems ranging from biological macromolecules in complex environments to molecular crystals in materials science. We investigated the nuclear relaxation of the TCNE:π:1ClN CT dimer in solvent near the Franck–Condon region. A novel theoretical approach based on *ab initio* molecular dynamics simulations in the DFT (TD-) framework and a multi resolution wavelet protocol has been applied to give a molecular picture of transient spectroscopic signals. A detailed study of vibrational modes and mode couplings allowed us to identify the main role played by some nuclear motions in non-radiative relaxation phenomena. Two low frequency vibrational modes and the central C=C stretching of TCNE can be considered as the key vibrational modes, which drive and modulate the relaxation to the ground electronic state.

This work is organized as follows: the first part is dedicated to the methodology used in this work for equilibrium and transient vibration analysis from *ab initio* molecular dynamics and the study of the main molecular factors involved in the ground state relaxation process. Section 3 is devoted to discussing the results. In Section 4, conclusions are given, and computational details used for modeling the system under study are given in the last section.

## 2 Methodology

### 2.1 Ground and excited state generalized normal mode definition

The theoretical grounds of the proposed strategy presented here is that at any temperature the so-called generalized normal-like modes correspond on average to uncorrelated momenta of atom groups.^72,73^*Via ab initio* molecular dynamics (AIMD) trajectories, generalized normal modes can be extracted and their power spectra (comparable to IR and Raman ones) can be computed by means of Fourier transform.^74–76^ In this section, we summarize in brief the protocol employed for the ground state and we refer the interested readers to the more detailed discussion of ref. 54. For each step of the trajectory, the rotational modes of the system have been projected out by a minimization procedure of the angular momentum with respect the orientation assumed by the molecule in the first time step. In this work, this procedure was applied only for the coordinates and momenta of TCNE rather than for the entire complex, since in this way an improved description (less noisy spectra and smoother peaks) of the vibrational mode composition and relative spectra was achieved. The generalized normal modes are computed by using the transformation matrix **L**, which diagonalizes the covariance matrix of the mass weighted atomic velocities **K**, with elements:1
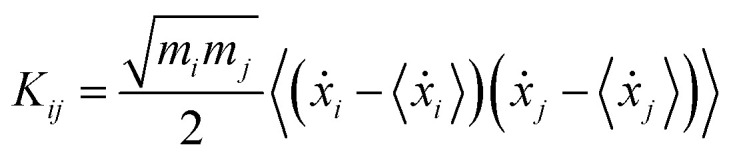
where *i* and *j* run over the 3*N* atomic Cartesian coordinates, and *m*_*i*_ is the mass associated with the corresponding cartesian velocity, *ẋ*_*j*_, collected along the trajectory. The quantity in 〈…〉 represents the ensemble average constructed in this work by averaging over the time. The normal modes have been obtained as the eigenvectors of **K** while the eigenvalues of **K** provide averaged kinetic energy for each mode.

An extension to the Franck–Condon region of the excited states of generalized normal mode and their relaxation dynamics of such a procedure were recently presented by some of the authors in ref. 77. According to this protocol, one or more excited state MD trajectories are collected first and then we assume that the normal modes are unchanged with respect to the ground state. This approximation allows projecting the excited state atomic velocities along ground state generalized normal mode vectors to obtain their evolution in the Franck–Condon region. Generalized mode velocities **Q̇** for the corresponding electronic state of interest are calculated at each time step by projecting either ground (GS) or excited state (ES) atomic velocities along generalized normal modes:

2 **Q̇**_GS/ES_(*t*) = **L**^†^*ẋ*_GS/ES_(*t*).

where **L** is the unitary transformation. which diagonalizes **K** and is assumed unchanged for both electronic states. The generalized normal mode velocity power spectrum *P*^*α*^(*ω*) for a given normal mode can be directly computed *via* the Fourier transform of their autocorrelation,^78^ according to the following expression:

3 *P*^*α*^(*ω*) = ∫〈*Q̇*^*α*^_GS/ES_(*τ*)*Q̇*^*α*^_GS/ES_(*t* + *τ*)〉_*τ*_e^−i*ωt*^d*t*

where *α* runs over the 3*N* generalized normal coordinates, 3 modes describe the translational and the other 3 modes account for the rotational collective motions, respectively.

### 2.2 Wavelet-based transient vibrational analysis

To elucidate the dynamic behaviour of atomic displacements during the nuclear motion, time correlation functions and Fourier analysis are suitable investigative tools that provide a time-averaged description of specific quantities in the frequency domain. Some theoretical approaches have been proposed over the years to preserve the time resolution in the vibrational analysis defining the vibrational modes as instantaneous^79–82^ or transient normal modes.^83,84^ Since we are interested in the molecular interpretation of a time evolving spectroscopic signal in the Franck–Condon region, a time resolved vibrational protocol was proposed in this work to analyze time dependent quantities extracted from *ab initio* molecular dynamics trajectories, relying on the continuous Wavelet Transform (cWT).^85^ At variance with other available techniques for signal processing, such as Fourier transform (FT) and short-time Fourier transform, the continuous wavelet transform allows for multi-resolution analysis enabling us to observe the temporal evolution of a vibrational feature. The cWT acts on a given time-dependent quantity *S*(*t*) in accordance with the relationship reported below:

4 *W*(*a*,*b*) = ∫*S*(*t*)*ψ*_*a*,*b*_(*t*)d*t*

The mathematical expression of a wavelet mother function is the following one:5
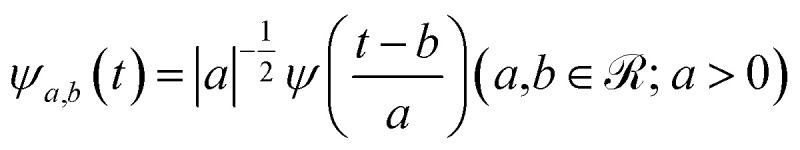



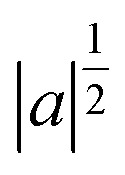
 is a normalization factor, *a* is the scale parameter, proportional to the inverse of frequency, and *b* is the translation parameter, which shifts the *ψ* function along the time axis for each value of *a*. By varying these two parameters, it is possible to analyze a given signal evolution extracted from AIMD simulations preserving the temporal information in addition to the frequency one. In the present work, we chose the Morlet wavelet as the mother function,^86^*ψ*, which provides optimal time-frequency resolution for the nature of our time-series according to previous studies.^54–59,87–90^ The time-dependent quantities (*S*(*t*) in eqn (4)) on which the wavelet transform is applied are the *Q̇*^*α*^_GS_ for the S_0_ ground state, the *Q̇*^*α*^_ES_ for the excited state or the time evolution of selected structural parameters extracted from the corresponding AIMD trajectory. The vibrational frequencies related to selected internal coordinates were also investigated by means of time-series Fourier transform. The cWT vibrational power spectra of signals computed from ground and first singlet excited state AIMD trajectories, the last ones conducted considering an average over the excited state trajectories, are presented as bidimensional (perspective) maps to facilitate reading. The time and frequency domains were reported in femtoseconds (fs) on the *x*-axis and in the wavenumber (cm^−1^) on the *y*-axis, respectively. The magnitude |*W*(*v*,*t*)|^2^ of the WT power spectrum is reported with a color scale (values reported in the figures). The frequency resolution ranges from ∼3 cm^−1^ in the out of plane bending region (below 200 cm^−1^), ∼10 cm^−1^ in the in plane CCN bending mode region (around 500 cm^−1^) and ∼30 cm^−1^ in the spectral region between 1450 and 1550 cm^−1^ (C=C stretching mode). The frequency values shown throughout the text for each vibrational mode were identified considering the maximum magnitude value of the signal.

### 2.3 Detection of vibrational couplings by the wavelet protocol

From a theoretical point of view, the canonical procedure for the resolution of the vibrational problem relies on the quantum mechanical picture of a stationary-state system in a minimum energy potential well, within the harmonic approximation (Hessian matrix diagonalization). Actually, the Hessian-based approaches, although very accurate, are not suitable for the vibrational analysis of large flexible systems especially if their realistic chemical environment is explicitly accounted for in the model. The potential energy surface (PES) of a chemical system is inherently anharmonic and several treatments can be exploited and implemented to account for higher terms in the potential energy expansion (*e.g.* variational or perturbative approaches) with a computational cost, which is at times prohibitive. Generalized normal modes extracted from the AIMD trajectory at finite temperature have been proven useful to naturally consider the anharmonicity of potential ruling the nuclear motion, providing couplings between modes and more accurate physics. Unlike time-independent signal processing techniques (*e.g.* Fourier transform), the wavelet protocol analysis untangle a signal in the time domain. Through the analysis of the temporal evolutions of generalized normal modes, **Q̇**(*t*), the time-resolved vibrational spectra are very informative about anharmonic vibrational couplings that occur on the excited state PES. The anharmonic coupling that we observe in the time-resolved vibrational spectra can be explained by considering two limiting examples. In particular, when a vibrational mode, named A, is anharmonically coupled to a different mode, say B, it implies that between them there is an exchange of kinetic energy and therefore that such oscillators are coupled. Considering the exemplary vibrational wavelet spectrum reported in Fig. 2, it can be observed that the magnitude associated with an excited-state vibrational mode shows an oscillating pattern with a Δ*t*-period. By slicing the main signal along the maximum of the magnitude and Fourier transforming the resulting trace the underlying modulating frequency is thus calculated. Alternatively, the anharmonic coupling can be revealed in the same time-resolved vibrational spectrum in which more than one signal associated with the main one is present in a different time-frequency region.

**Fig. 1:**
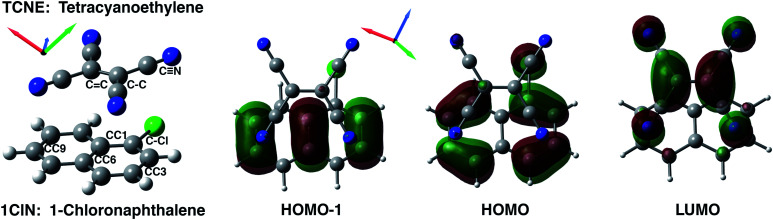
TCNE:π:1ClN CT complex (left) and top view of frontier molecular orbitals (isovalue: 0.03) mainly involved in the S_1_ ← S_0_ and S_2_ ← S_0_ electronic transitions. From left to right, HOMO−1, HOMO and LUMO contour plots computed for a minimum energy structure in DCM solvent. Carbons are reported in gray, hydrogens in white, blue is for nitrogens and green for chlorine atoms.

**Fig. 2:**
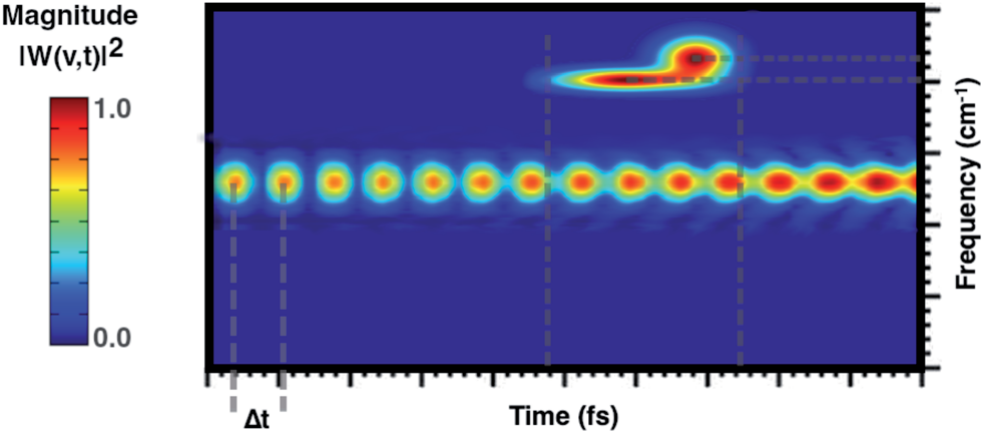
Exemplary wavelet perspective map that shows the vibrational coupling found following the wavelet analysis of generalized normal modes extracted from an *ab initio* molecular dynamics trajectory. The time domain is reported along the *x*-axis, the frequency range along the *y*-axis and the color scale states for the magnitude |*W*(*v*,*t*)|^2^. Vertical dashed gray lines identify the Δ*t*-period of the oscillation pattern and the temporal range where the anharmonically coupled signal appears. Horizontal dashed gray lines identify the frequency values associated with the coupled signal.

### 2.4 Nonadiabatic coupling matrix elements

To better analyze relaxation pathways upon excitation, first order Cartesian nonadiabatic coupling matrix elements (NACs, eqn (6)) were computed analytically in the framework of linear response TD-DFT^91,92^ on structures regularly displaced along selected generalized normal modes, *Q*^*α*^, extracted from the *ab initio* molecular dynamics trajectory:6
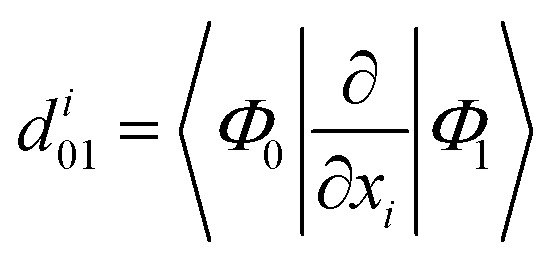
in which *x*_*i*_ is an atomic Cartesian nuclear coordinate, and |*Φ*_0_〉 and |*Φ*_1_〉 are the ground and first excited electronic states, respectively. In addition, NACs along a low frequency vibrational mode obtained by diagonalizing the geometrical hessian matrix of a minimum energy structure were computed to unveil the role played by the intermolecular plane distance in the relaxation process. As for all DFT methods, the computation of excited state properties can be obtained from the density and transition density (*via* time dependent theory). Given the recent implementation of analytic NACs,^91,92^ this computation has been made more affordable, adding just a little extra cost with respect to the computation of the geometric gradient for excited states.

From eqn (6), the derivative couplings *d*^*i*^_01_ will be large near surface crossing regions and will tend to infinity when approaching a conical intersection. In what follows we will report and discuss the Frobenius norm of the NAC matrix and the TD-DFT energy scan for each geometry displaced along the corresponding normal mode.

## 3 Results and discussion

The equilibrium ground state CT complex molecular dynamics show the π–π stacked conformation in which the two monomers always interact with the same initial molecular side, although TCNE slides back and forth between the two aromatic rings of 1ClN. The two monomers assume large mutual displacements around an average value of the center of mass (COM) distance of 3.7 Å ± 0.2. On the other hand, in the excited state trajectories, the two units tend to be closer (−0.1 Å) due to the increased partial charge on the two subunits, which enhanced the coulombic attraction between them. The photoinduced CT leads to structural reorganization involving both subunits which can be summarized as follows: concerning the 1ClN donor monomer the C–Cl distance undergoes a shortening (−0.05 Å), when in the excited state, and an alternation of the C–C bond lengths also occurs in accordance with the HOMO−1 and LUMO spatial extents, depicted in Fig. 1. For the TCNE acceptor, the central C=C bond is stretched out (about 0.06 Å), with a concomitant and symmetric stiffening (−0.02 Å) of the four (=C–)C–C(≡N) distances. In contrast, the bond lengths of the four cyano groups actually do not change when passing from the ground to the excited electronic state.

### 3.1 Excited state transient vibrational analysis

We focused the time-resolved vibrational study mainly on three significant vibrational modes, localized on the TCNE acceptor subunit. In detail, these modes are the central C=C stretching, the out-of-plane bending in which the cyano C≡N groups approach the 1ClN and the symmetric in-plane deformation of the four CCN angles. The C=C stretching mode, being fully spatially localized, is strictly related to the C–C distance and its bond order, which are both highly sensitive to the electronic density rearrangement upon excitation (*i.e.* charge transfer and coulombic interactions). The composition of the three generalized normal modes of interest extracted from the ground and the excited state AIMD simulations is reported in Fig. 4 to 6, along with the corresponding time-resolved wavelet spectra (for completeness, the time independent FT spectra are reported in Fig. S2, S4 and S6 in the ESI†). Their time evolution has been recently characterized with high time and frequency resolution by Femtosecond Stimulated Raman Spectroscopy (FSRS) by Mathies and co-workers,^16^ providing a prominent spectroscopic study, briefly summarized below. The steady-state UV-Vis absorption spectrum of the TCNE:1ClN CT complex recorded in DCM solution shows two CT bands (3.04 eV and 2.31 eV) corresponding to different contributions of π symmetry orbitals. The ground state Raman spectrum of the neutral TCNE shows a peak centered at 1565 cm^−1^. In contrast, the chemically reduced TCNE, used as a reference for the photoexcited one, shows two red-shifted peaks at 1390 and 1421 cm^−1^ depending on the distance of the counterion. The analysis of the transient absorption spectrum revealed that the excited state absorption signal decays with a time constant of 5.9 ± 0.2 ps, which corresponds to the charge recombination of the biradical species. The stimulated emission band is dominated by two main frequencies, the excited state TCNE out of plane bending at 153 cm^−1^ and a bending mode of the DCM solvent (278 cm^−1^). Additionally, in time resolved FSRS, they found peaks corresponding to fundamentals and overtones or their combination bands in the frequency domain. Focusing on fundamentals, the narrow band at 534 cm^−1^ (fwhm ∼ 13 cm^−1^) was ascribed to an in plane bending of the C≡N groups and the broader peak at 1392 cm^−1^ should correspond to the C=C double bond stretching of the TCNE radical anion. The sensitivity of this mode to the random orientations of the TCNE:1ClN complex could justify the observed broader peak (fwhm ∼ 55 cm^−1^) with respect to the in plane bending.

**Fig. 3:**
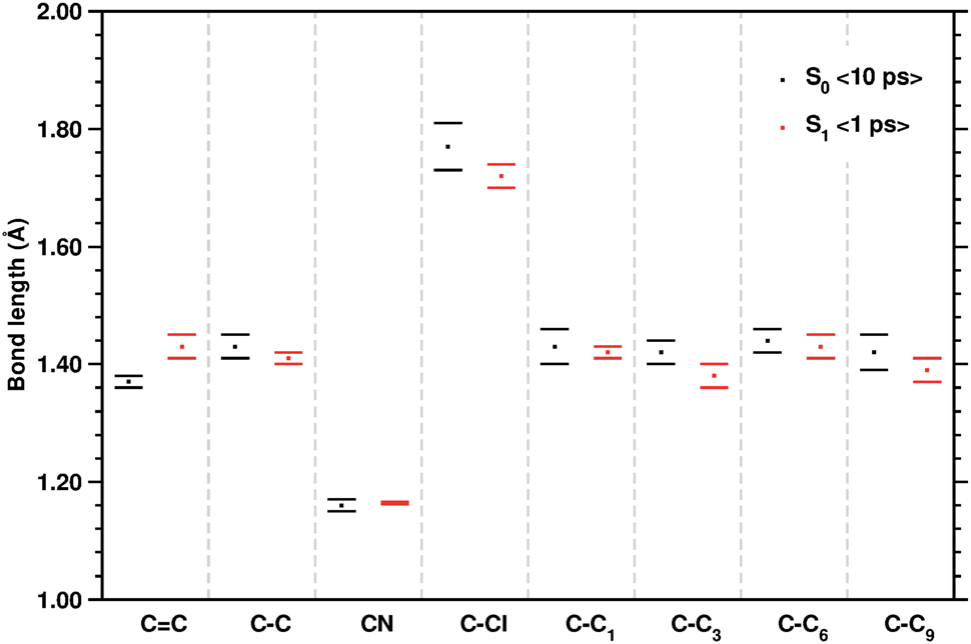
Mean values of the selected structural parameters of the TCNE:1ClN CT complex, extracted from 10 ps S_0_ and the last 1 ps S_1_ AIMD trajectories. Labels are referred to Fig. 1, bond lengths are reported in Angstroms (Å), and standard deviations are given as error bars.

**Fig. 4:**
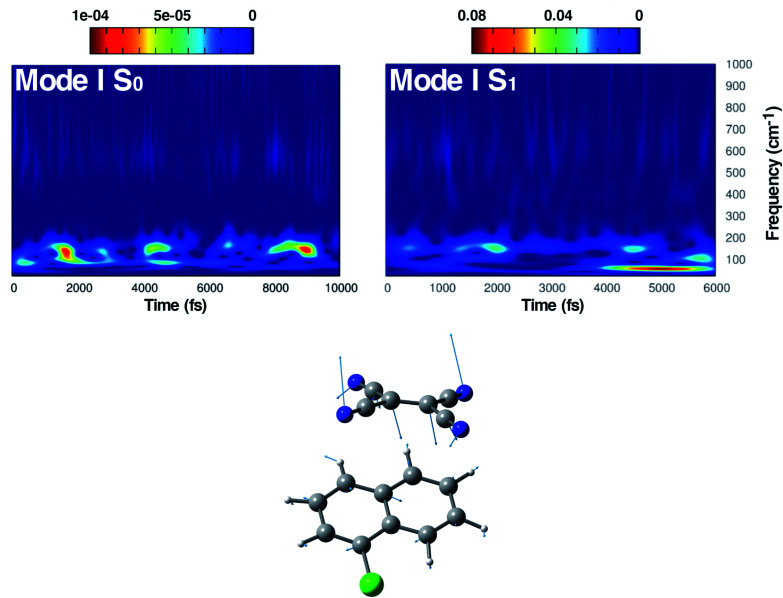
Time resolved wavelet spectra of the out of plane bending mode (Mode I) extracted from the ground (left, 10 ps) and the excited state (right, 6 ps) trajectory. The magnitude |*W*(*v*,*t*)|^2^ of the wavelet power spectrum is reported as a color scale on the top.

**Fig. 5:**
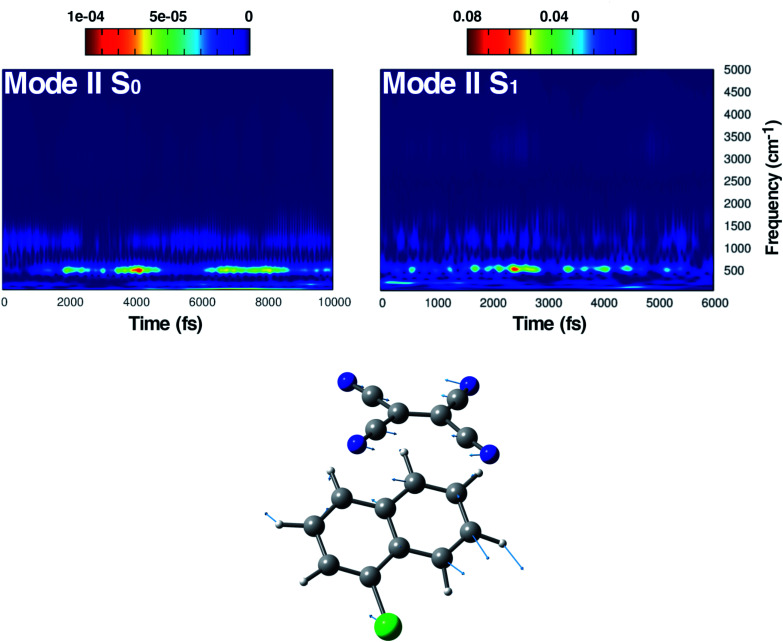
Time resolved wavelet spectra of the symmetric in plane CCN bending (Mode II) extracted from the ground (left, 10 ps) and the excited state (right, 6 ps) trajectory. The magnitude |*W*(*v*,*t*)|^2^ of the wavelet power spectrum is reported as a color scale on the top.

**Fig. 6:**
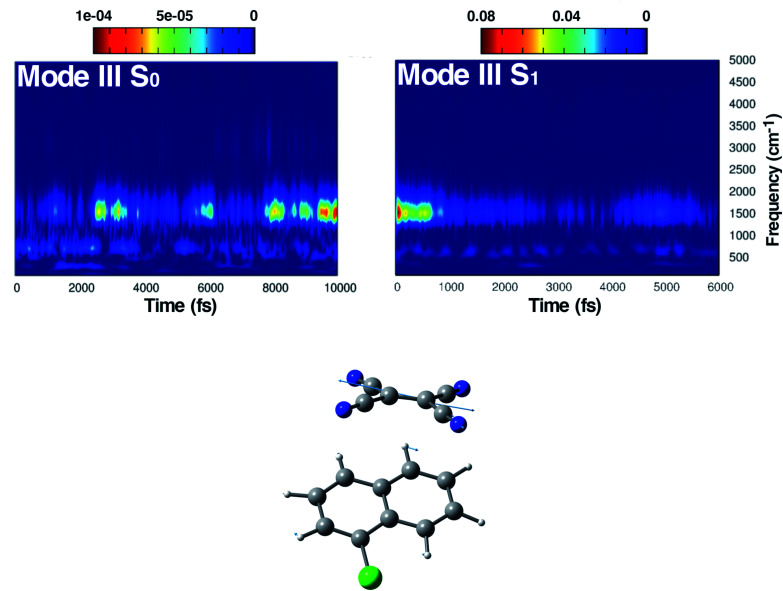
Time resolved wavelet spectra of the central C=C stretching (Mode III) extracted from the ground (left, 10 ps) and the excited state (right, 6 ps) trajectory. The magnitude |*W*(*v*,*t*)|^2^ of the wavelet power spectrum is reported as a color scale on the top.

Here we present our time-resolved vibrational analysis focusing on the relaxation of these modes in the Franck–Condon region. The analysis of the ground state vibrational modes, used as a reference, has not been addressed intensively. This interesting aspect has been analyzed in a previous study of some of the authors^54^ for a model peptide in water solution.

The first vibrational mode discussed here can be described in both electronic states as an out-of-plane bending involving all cyano groups in a synchronous motion towards the 1ClN donor monomer. The spectrum on the left of Fig. 4 presents a main contribution centered in the S_0_ at 160 cm^−1^. This mode appears to be highly coupled with another collective low frequency motion of the two monomers approaching closer to each other (at ∼90 cm^−1^, assigned with the help of Hessian-based vibrational analysis, data not shown). These low frequency coupled motions can be very important in modulating the charge recombination event in the excited state dynamics since their ability of narrowing the intermolecular distance, thus promoting a better overlap of the frontier orbitals. The out-of-plane bending mode frequency was found to be unaffected by the excitation (157 cm^−1^ main peak of Fig. 4, right panel), showing a excellent agreement with the experimental value (153 cm^−1^)^16^ and a different frequency modulation over time can be observed. The frequency modulation over time has been analyzed for both S_0_ and S_1_ but it did not lead to a clear coupling with other vibrational modes. In contrast, this mode is still coupled with other low frequency motions (harmonic Hessian-based values at 54 and 65 cm^−1^, graphical representations of these modes are reported in Fig. S3 in the ESI†), representing also this time collective motions promoting the rigid approaching of the two molecular planes to one another. Interestingly these modes, leading presumably to charge recombination and therefore non-radiative decay, gain contribution not immediately but after a few initial ps from excitation. This is in accordance with the relatively long experimentally observed life time, *i.e.* the 5.9 ± 0.2 ps decay time of the excited state transient absorption signal.^16^ The Raman activity of the CT complex is dominated by this vibrational coupling between the out of plane bending mode of the acceptor monomer (TCNE) and low frequency vibrational modes affecting both monomers. The activity of the latter could be decisive in the relaxation of the excited state to the ground state with consequent recombination of the charge. We later demonstrate that the rising and decay times of modes below 100 cm^−1^ are consistent with the observed decay time of transient absorption experiments.

In Fig. 5, the time-resolved vibrational analysis in both electronic states of the vibrational mode involving the in phase symmetric CCN bending of the four groups on the TCNE molecule is discussed next. In the ground state a well-resolved peak is observed at 538 cm^−1^. Upon excitation this mode is present at a slightly blue-shifted value with respect to the ground state (545 cm^−1^, −7 cm^−1^) and also this time is in good agreement with FSRS experimental findings (534 cm^−1^).^16^ The analysis of the magnitude oscillations in the wavelet spectra of the CCN bending mode allowed us to find some couplings with low frequency vibrational modes. The magnitude modulations over time have been extracted and Fourier transformed for both ground and excited state signals. In Fig. S5 in the ESI† we report the analyses in the ground (left panel) and in the excited state (right panel) in which a clear modulation is presented by several anharmonically coupled low frequency modes (24, 47, 60, 150 cm^−1^) representing collective vibrational modes that involve both monomers such as dimer breathing, mutual rotations and sliding of molecular planes.^93,94^

The last vibrational mode discussed here is shown in Fig. 6 and concerns the C=C stretching which is completely localized on the TCNE subunit. This mode shows a well defined feature in the ground state centered at 1530 cm^−1^ that is coupled with a less intense signal at 691 cm^−1^, the latter presumably belonging to an out of phase distortion of the TCNE molecule as suggested by the Hessian-based vibrational analysis used as a reference. In a previous experimental study based on spontaneous Raman spectroscopy,^16^ the C=C stretching of neat TCNE in acetonitrile solution is assigned to a peak centered at 1565 cm^−1^. Following the excitation to the S_1_ electronic state, the TCNE monomer receives electronic density promoting an elongation of the central double bond, the vibrational mode becomes localized only on the acceptor molecule, and thus the WT spectrum mainly consists of a well resolved and isolated peak at 1485 cm^−1^. This peak is red shifted (−45 cm^−1^), as expected, with respect to S_0_ and the anharmonic coupling with the lowest frequency mode is now less important. This trend of the frequency values can also be rationalized considering the lowering of the C=C bond force constant following the excitation, as can be observed by inspecting the mean value of the double bond distance, computed as an average of AIMD trajectories in the excited state, see Fig. 3 (corresponding values are reported in Table S1 in the ESI†), which differs by about +0.06 Å with respect to the ground state value (1.37 Å). Actually the 1485 cm^−1^ frequency value assigned by us to the C=C stretching does not correspond to the attribution based on the chemically reduced TCNE spontaneous Raman spectrum bands.^16^ See the next section for further discussion.

### 3.2 Refining vibrational frequency assignments

**Fig. 7:**
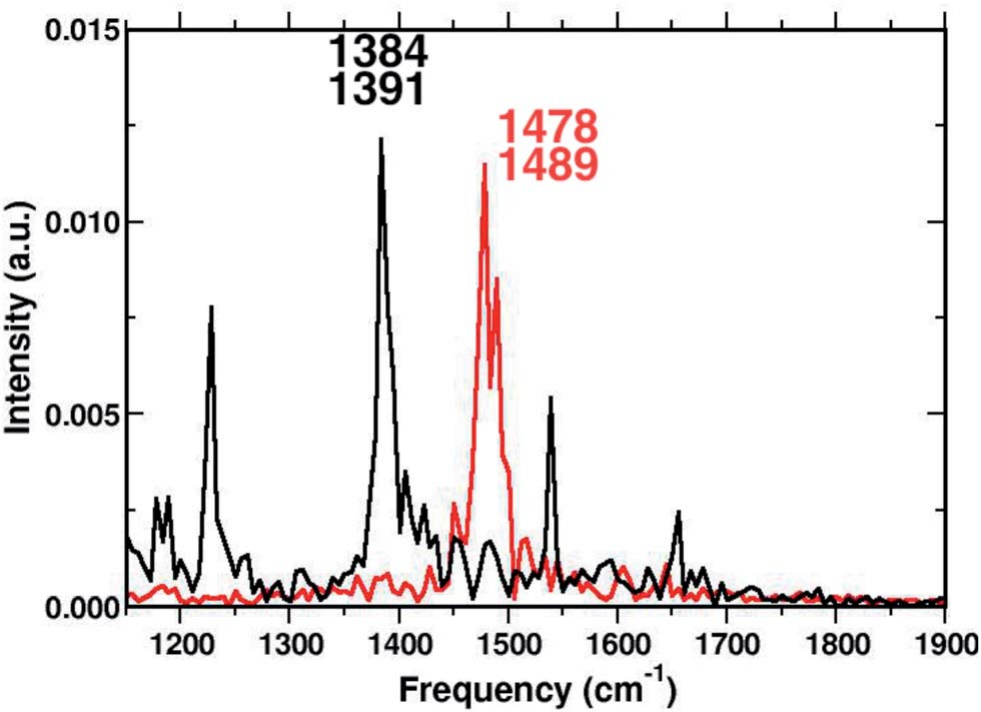
Time-independent spectrum in the 1150–1900 cm^−1^ spectral region, computed by the Fourier transform of the CC1 (black trace) and C=C (red trace) bond lengths averaged from the excited state AIMD trajectories.

To corroborate our assignment of the C=C stretching mode, we analyzed the temporal evolution of the structural parameters of both subunits in terms of frequency content. This approach led us to the accurate and unambiguous identification of the molecular motion from which the vibrational bands, observed by the excited state dynamics, originate. In Fig. 7 we report the vibrational spectrum along the CC1 internal coordinate of the 1ClN monomer as well as the C=C coordinate of the TCNE molecule. Computed average values (see CC1 and C=C reported in Fig. 3) along excited state trajectories are in agreement with the excited state energy minima of CT complexes (*i.e.* 1.421 Å for CC1). The Fourier transform of these oscillating signals showed the vibrational frequencies associated, and the CC1 distance showed a main peak centered at 1384 cm^−1^ with a weak shoulder at 1391 cm^−1^, while the C=C frequencies were found at 1478 and 1489 cm^−1^. Furthermore in Fig. 7, it is clear that there are no mutual overlaps of the two main bands. In the frequency range of 1380–1480 cm^−1^ the TCNE has no active normal modes of vibration as it was additionally verified by the TD-DFT frequency calculations conducted on the fully optimized TCNE:1ClN excited state structures (data not shown). This further supports the absence of modes ascribed to the TCNE in this spectral region as already shown in the previous section.

To have a clearer picture of the vibrational modes involving mostly the 1ClN monomer, it was possible to extract the generalized normal modes considering only the 1ClN subsystem from both ground and first singlet excited state molecular dynamics (the corresponding time independent FT and time-resolved wavelet spectra are reported in Fig. S7 and S8 in the ESI†), neglecting those related to the TCNE subunit. As a main result we were able to individuate and assign for the first time the vibrational feature experimentally observed at 1392 cm^−1^ to the CC1 stretching mode, localized on the 1ClN molecule. Moreover, this result is in accordance to the fact that under this experimentally broad spectral feature (fwhm = 55 cm^−1^)^16^ the CC stretching modes of naphthalene moieties are present. Upon excitation, we found two main isolated and well resolved vibrational features at 1392 (weaker) and 1428 cm^−1^, where the CC1 stretching played a major role from our analysis. These two values were blue shifted by 16 and 48 cm^−1^, respectively, compared to those computed in the S_0_ electronic state. This last trend of frequencies can also be rationalized in light of the CC1 structural rearrangements reported in Fig. 3.

### 3.3 Molecular factors leading to non-radiative relaxation paths

**Fig. 8:**
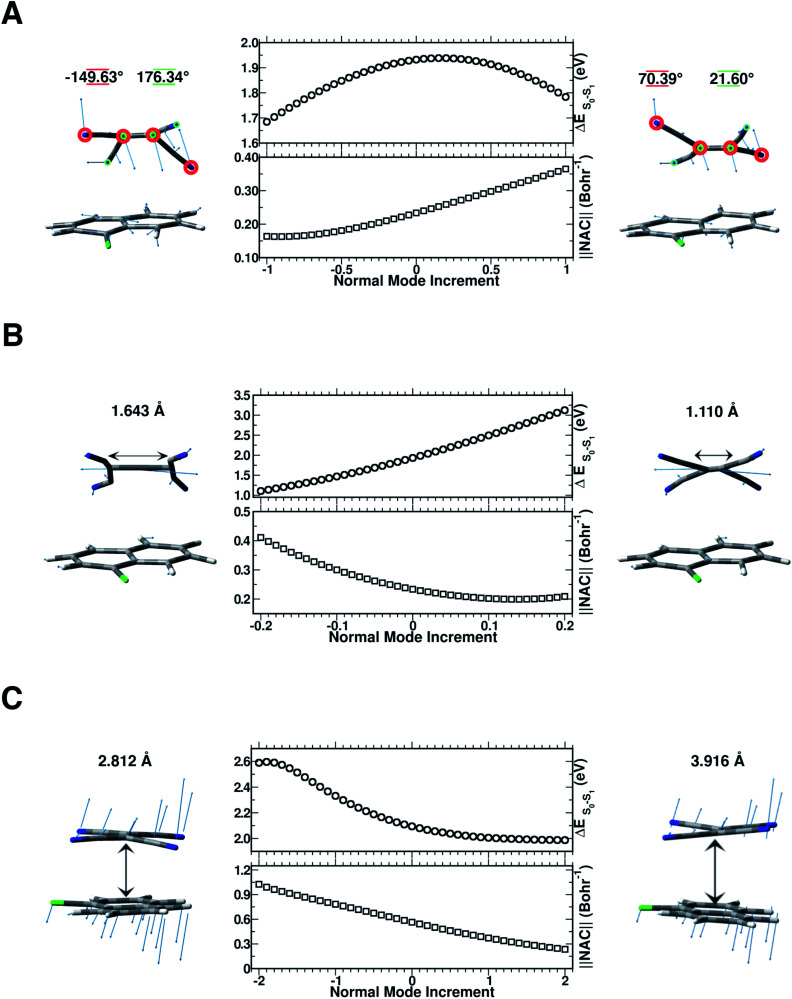
Panel A, TD-DFT energy (eV) computed for each *δ* increment for the S_1_ ← S_0_ electronic transition (center top). Frobenius norm of the nonadiabatic coupling matrix computed along with the out-of-plane TCNE bending mode increased by *δ* = ±0.05 equal to a deformation of the N–C=C–N dihedral angle of ∼0.65° (center bottom). Panel B, TD-DFT energy (eV) computed for each *δ* increment for the S_1_ ← S_0_ electronic transition (center top). Frobenius norm of the nonadiabatic coupling matrix computed along with the C=C stretching mode increased by *δ* = ±0.01 equal to a deformation of the C=C bond length of ∼0.01 Å (center bottom). Panel C, TD-DFT energy (eV) computed for each *δ* increment for the S_1_ ← S_0_ electronic transition (center top). Frobenius norm of the nonadiabatic coupling matrix computed along with a low frequency collective mode (at 85 cm^−1^) increased by *δ* = ±0.1 equal to a change of COM distance of ∼0.033 Å (center bottom). To the sides of each plot is reported the maximum displacements with respect to the equilibrium position of the geometries considered during the normal mode coordinate scan. In Panel A, the numerical values underlined in red and green refer to the NC=CN dihedral angles enclosed in red and green circles, respectively. In panels B and C, the C=C bond length and the center of mass distance between the two subunits are shown above each corresponding mode, respectively. For positive *δ* values the structural displacement follows the direction of the normal mode vectors (blue arrows). In the case of the C=C double bond, due to the vector orientation the positive *δ* displacements shorten the bond distance, *vice versa* for negative displacements.

In this section, we discuss the possible molecular factors that can contribute to the activation of non-radiative relaxation channels of the CT complex from the first singlet excited state to the ground state one. The temporal evolution of selected structural parameters has been extracted from the AIMD simulations and primarily investigated by means of their frequency contents. The previous analysis of vibrational frequencies allowed us to identify the main candidate vibrational modes most likely involved in the non-radiative decay: the out-of-plane bending presumably responsible for the back electron transfer event (Fig. 8 Panel A), the central C=C stretching mode (Fig. 8 Panel B) and the low frequency collective mode (Fig. 8 Panel C), which rigidly approaches the two subunits one another. To check our hypothesis both TD-DFT energy scans and first-order NAC element matrix evaluation were performed for each identified vibrational mode according to the procedure presented in the Computational details. In Fig. S9 in the ESI,† the FT of the NC=CN dihedral angle time evolution is reported, showing a clear feature at about 160 cm^−1^, easily assignable to the out of plane bending mode of the TCNE monomer. Although there is a symmetrical distortion during this motion, it should be noted that with positive *δ* value increments, the cyano groups move away from the 1ClN, while the C=C region tends to approach the donor, accounting for the bell shape of the vertical excitation energies calculated along this coordinate. These excitation energies are not the only factors to consider and we analyzed next the NACs. The corresponding one evaluated along the out of plane bending mode coordinate (Fig. 8 Panel A) showed an anticorrelated trend between the highest NAC value (0.365 bohr^−1^) and lowest TD-DFT excitation energy (0.150 eV lower with respect to the initial geometry) reached when the four cyano groups bend outward the 1ClN unit during the nuclear motion. These analyses unveil the high involvement of this motion in the non-radiative pathway.

**Fig. 9:**
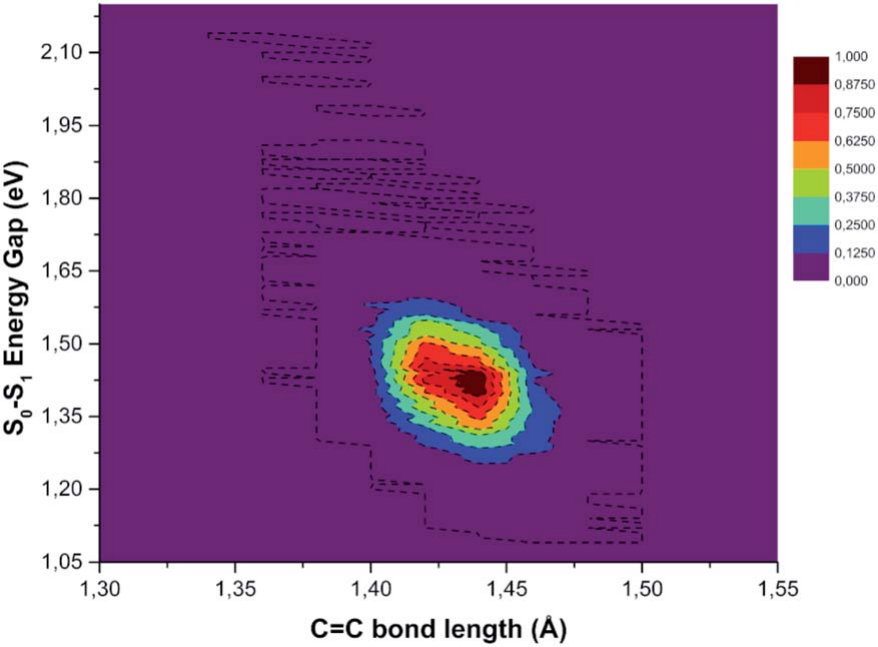
Normalized distribution of the central C=C bond length of the TCNE unit (abscissa axis) extracted from excited state AIMD and the electronic energy gap (ordinate axis) values between ground and first singlet excited states.

In Fig. S10 in the ESI† the FT of the C=C bond length temporal evolution shows a clear peak centered at 1478 and 1489 cm^−1^. TD-DFT excitation energy scans and NAC norm evaluation along the C=C normal coordinate are thus performed and reported in Fig. 8 Panel B, respectively. When the two carbon atoms approach one another till the closest scanned distance, the TD-DFT excitation energy value reaches its maximum value (+1.192 eV with respect to the starting geometry at 1.932 eV) and the NAC trend goes in the opposite direction (up to 0.209 bohr^−1^). At the largest C=C distance, the TD-DFT value decreases (−0.827 eV), while NACs reach their maximum value with a high positive slope (up to 0.411 bohr^−1^). These data suggest that the C=C double bond may be a very sensitive probe of the photoinduced charge transfer extent as well as being the most important molecular factor affecting non-radiative relaxation. In this regard, we analyzed the normalized distribution between the TCNE C=C bond length and S_0_–S_1_ energy gap values obtained by averaging the excited state trajectories in Fig. 9. When the bond length increases, reaching its maximum value, the energy gap of the two electronic states shows an opposite behaviour. According to this, presumably the C=C distance in the excited state mainly affects the adiabaticity of the two involved potential energy surfaces providing a channel to non-radiative molecular relaxation of the entire TCNE:1ClN CT complex towards the ground state. The computed distribution of C=C values agrees with that obtained by X-ray crystallographic studies and Raman spectra of the TCNE^*n*^ [*n* = 0, −1, −2] CT salts.^95^ The important role of C=C elongation has been previously highlighted by high-precision coupled cluster calculations.^96^

To investigate the role of the mutual TCNE:1ClN approach in the non-radiative relaxation pathway, we report in Fig. S11 in the ESI† the distance between the two centers of mass of the two molecules along with its Fourier transform. This distance showed a significant decrease as a consequence of electronic density redistribution following the photoexcitation (∼−0.064 Å on average). This analysis highlighted the presence of low-frequency vibrational modes mainly in the 25–90 cm^−1^ spectral range. In Fig. 8 Panel C, the TD-DFT excitation energy and NAC values computed for each displacement along this low frequency mode showed that the closeness of the two monomers increases both the excitation energy (0.495 eV with respect to the starting geometry at 2.095 eV) and NAC norm (1.026 bohr^−1^). On the other hand, when the interplanar distance between the two subunits increases a smooth lowering of excitation energy is observed while the NAC values decrease with a larger negative slope. The distribution in contrast did not highlight a correlation between the average of COM distances and the S_0_–S_1_ energetic gap; please see the Fig. S12 in the ESI.† The average values of the structural parameters extracted from the excited state molecular dynamics simulations (reported in Fig. 3 and S9–S11 in the ESI†) correspond to normal mode displacements *δ* of 0.5 for the out of plane bending mode (Fig. 8, Panel A), −0.05 for the C=C stretching (Fig. 8, Panel B) and 0.7 for the low frequency collective mode (Fig. 8, Panel C). The corresponding NAC magnitude is lower compared to the whole range investigated in the normal coordinate scan.

In summary, the out of plane bending, the central C=C stretching and the subunits mutual displacements have been highlighted to participate in non-radiative relaxation phenomena and the correlated vibrational modes are showed to affect the energy gap between the two electronic states. The geometrical deformations conducted along the generalized normal mode coordinate which mostly contribute to the excited state relaxation are reflected mainly in high values of the non-adiabatic coupling elements and low energy gaps. The characteristic trends observed for C=C stretching mode suggest that it may be, predominantly, a key vibrational mode in the return to the ground electronic state.

## 4 Conclusions

In the present study, a prototypical spatially separated molecular system has been investigated in terms of adiabatic molecular dynamics simulation in implicit solvent in both ground and first singlet excited states which showed a strong charge transfer character. We focused on the nuclear relaxation upon photoexcitation downhill the Franck–Condon region with the aim to: (1) unveil the role of key vibrational modes and modes couplings; (2) correlate some vibrational fingerprints with electronic properties; (3) explain at the molecular level the excited state Raman activity provided by time-resolved vibrational spectroscopy of a challenging system to model.

In reference to the methodology used in this work, to define the excited state vibrational modes we used a strategy based on employing the ground state generalized normal modes as a basis for projecting the excited state mass weighted nuclear velocities. Moreover, the data processing entrusted to the multi-resolution wavelet protocol, in addition to the precise identification of the main vibrational modes and the associated anharmonic frequencies, allowed us to observe their temporal evolution and dynamical vibrational couplings. From a careful analysis of the oscillatory components of such features, the vibrational couplings of the in- and out-of-plane bending modes of the TCNE acceptor with collective low frequency modes have been discovered. In this work we were able to reproduce and interpret experimental FSRS experiments from Mathies and coworkers.^16^ In this regard, the computed frequency values of the out of plane bending at 157 cm^−1^ and the CCN in plane bending at 538 cm^−1^ in excellent agreement with experimentally observed ones at 153 and 534 cm^−1^, respectively. In addition, further insights can be deduced from the rise and decay times of low frequency vibrational modes coupled to the out of plane bending which are consistent with the excited state absorption signal decays (5.9 ± 0.2 ps) experimentally observed in the same work. In our opinion, this highlighted pathway could be decisive in the relaxation of the excited state to the ground state with the consequent recombination of the charge. From a methodological point of view, to carry out a formal kinetic study to extract the CT lifetime, which is not the aim of the present work, non-adiabatic approaches are needed. The vibrational analysis beyond the harmonic regime and the possibility of exploiting atomistic details provided by the AIMD simulations has also shown its potentiality allowing us to assign correctly the 1392 cm^−1^ band to the CC stretching of the 1ClN donor monomer initially ascribed by experimentalists to the C=C stretching mode of the TCNE. TD-DFT energy scans and first-order NAC element matrix evaluation performed on nuclear geometries displaced along generalized normal modes unveiled the main molecular factors that contribute to the activation of non-radiative relaxation channels of the CT complex. In more detail, the central C=C stretching mode is the key vibrational mode involved in the non-radiative pathway towards the ground electronic state. The detailed understanding on the molecular scale of photophysics and photoreactivities in the excited electronic states can be used to guide and modulate photoinduced processes and the future rational design of photosensitive materials and sensors.

## 5 Computational details

All presented calculations were performed by using the Gaussian electronic structure software package.^97^ The ground state energies, gradients and higher order energy derivatives of some conformers of the TCNE:π:1ClN CT complexes were obtained using the hybrid Becke, 3-parameter, Lee–Yang–Parr (B3LYP) density functional^98–100^ with a 6-31+G(d,p) basis set. Weak dispersion forces between the two subunits were described correcting potentials with Grimme's dispersion (GD3).^101–106^ Time-Dependent Density Functional Theory (TD-DFT), within the linear response formalism, using the range-separated version of the hybrid B3LYP density functional with the Coulomb-attenuating approach (CAM-B3LYP)^107^ also this time using 6-31+G(d,p) and GD3, was instead employed to describe accurately excited states. The long-range corrected Coulomb attenuated hybrid functionals class^107–111^ has been shown to predict charge transfer and Rydberg-like excitation energies/bands more accurately with respect to hybrid density functionals^112–120^ (*i.e.* B3LYP, Perdew–Burke–Ernzerhof-0 (ref. ^121^ and ^122^)). Additionally, we accounted for dichloromethane (ε = 8.93, DCM) solvation effects *via* a polarizable continuum model in its conductor like version (C-PCM).^123–128^ Solvent response in the excited state was treated using an available linear-response non-equilibrium formalism. We checked that at least in the initial excited state starting configurations the solvent response in term of average (polarized solute)-solvent energies is ∼13/15 kcal mol^−1^ if computed with State-Specific (SS) solvation.^129,130^ The state specific solvent response can be very important for kinetics studies in the subfemtosecond regime of relaxation. On the other hand, given the additional extra cost of SS solvation that has to be repeated at each step of the dynamics, we chose to adopt the standard non-equilibrium linear response for the solvent in the excited states also because in our opinion this approximation does not substantially affect the observed overall relaxation mechanism.

The amount of charge transferred between the two TCNE and 1ClN fragments in both ground and first singlet excited states was quantified by employing the Natural Bond Orbitals (NBO) charge partitioning scheme,^131–136^ along with calculating the value of the density based CT index (*D*_ct_), introduced by Ciofini and coworkers.^137–141^ Such detailed structural analysis of some conformers also in terms of relative stability, and ground and first singlet excited state vibrational characterization, namely Hessian-based vibrational analysis, are currently under investigation.

### 5.1 Ground and excited state *ab initio* molecular dynamics sampling

Ground state *ab initio* molecular dynamics (AIMD) simulation according to the Atom-centered Density Matrix Propagation (ADMP) formalism^67–71^ was performed for 10 ps, after 1 ps of equilibration, using a time step of 0.2 fs. The B3LYP/6-31G(d,p)/DCM(C-PCM)/GD3 theory level was used and a temperature of 300 K was enforced by scaling regularly nuclear velocities. Three distinct excited state AIMD simulations (Born-Oppenheimer Molecular Dynamics, BOMD) were performed for about 6 ps each, with a time step of 0.7 fs according to the TDCAMB3LYP/6-31G(d,p)/DCM(C-PCM)/GD3 potential. From the ground state phase space sampling, we chose 3 points as starting configurations and momenta of as many excited state MDs. The starting points were picked up to reproduce some crucial structural and electronic characteristics such as: (i) the S_1_ ← S_0_ and S_2_ ← S_0_ energy separations, (ii) the amount of CT character of the resulting S_1_ excitation in these configurations, (iii) and the distances of the center of mass of the two units (the starting geometries including an analysis of such parameters are reported in Fig. S1 in the ESI†). To preserve accuracy in the subsequent time resolved vibrational analysis, all the AIMD trajectories have been performed in a microcanonical (NVE) ensemble. Harmonic frequency calculations performed on the same ground state minimum energy structure ensure that the presence or the absence of diffuse s and p functions in the basis sets leads to practically overlapping results and that the maximum deviation was about 10 cm^−1^ on a high frequency mode, see Fig. S13 in the ESI.† Initial geometries were chosen from the ground state trajectory checking several properties such as: the accuracy in the prediction of S_0_–S_1_ excitation with respect to the experimental value, the amount of CT (by inspecting the NBO charges), and the distances of the center of mass of the two units. TD-DFT single point calculations were also performed for twenty configurations extracted regularly every 500 fs from the ground state trajectory to better characterize the average vertical excitation energies and electronic density redistribution represented by NBO charges upon the excitation for both S_1_ ← S_0_ and S_2_ ← S_0_ transitions along the trajectory (see Fig. S14 and S15 in the ESI,† respectively). In this way, both a consistent charge transfer of the low lying transition and a significant energy separation between the two excited states were checked on average along ground state dynamics.

## Author contributions

FC, PC, AP, and NR: project. FC, PC: data collections. FC, PC, UR and MGC: data analysis. All authors interpretation of data and writing.

## Conflicts of interest

There are no conflicts of interest to declare.

## Supplementary Material

SC-012-D1SC01238J-s001
