# Supplementary material for: Exploring the Franck–Condon region of a photoexcited charge transfer complex in solution to interpret femtosecond stimulated Raman spectroscopy: excited state electronic structure methods to unveil non-radiative pathways
Source: Chem Sci. 2021 Apr 28;12(23):8058–72. doi: 10.1039/d1sc01238j (PMC8208128; doi:10.1039/d1sc01238j)
Supplement: SC-012-D1SC01238J-s001 [file SC-012-D1SC01238J-s001.pdf]

Electronic Supplementary Material (ESI) for Chemical Science.
This journal is © The Royal Society of Chemistry 2021

Electronic Supporting Information Exploring the Franck-Condon region of a photoexcited charge transfer complex in solution to interpret femtosecond stimulated Raman spectroscopy: excited state electronic structure methods to unveil non-radiative pathways

Federico Coppola,[a,‡] Paola Cimino,[b,‡] Umberto Raucci,[a] Maria Gabriella Chiariello,[a] Alessio Petrone*[a] and Nadia Rega,*[a,c]

E-mail:

a) Department of Chemical Sciences University of Napoli Federico II, Complesso Universitario di M.S. Angelo, via Cintia, Napoli 80126, Italy

b) Department of Pharmaceutical Sciences, University of Salerno, Salerno 84084, Italy

c) CRIB, Centro Interdipartimentale di Ricerca sui Biomateriali, Piazzale Tecchio, I-80125, Napoli, Italy

‡ These authors contributed equally to this work

Correspondence to:

Alessio Petrone, alessio.petrone@unina.it

Nadia Rega, nadia.rega@unina.it

Contents

Figure S1: Initial geometries to run excited state BOMD simulations, extracted from ground state AIMD simulations.

Table S1: Mean values of selected structural parameters extracted from S_0 and S_1 *AIMD* of TCNE:1ClN CT complex.

Figure S2: Ground and excited state time-independent Fourier spectra of the out of plane bending mode.

Figure S3: Composition of two low frequency normal modes observed in the excited state Wavelet spectrum of the out of plane bending mode.

Figure S4: Ground and excited state time-independent Fourier spectra of the in plane bending mode.

Figure S5: Fourier Transform analysis of the oscillating behaviour observed in the in plane CCN bending Wavelet spectra.

Figure S6: Ground and excited state time-independent Fourier spectra of the central C=C stetching mode.

Figure S7: Ground and excited state time-independent Fourier spectra of the CC stretching mode.

Figure S8: Generalized normal mode composition of the CC1 stretching mode along with ground and excited state time-dependent Wavelet spectra.

Figure S9: Temporal evolution of the two NC=CN dihedral angles extracted from the excited state AIMD trajectories along with its Fourier Transform analyis.

Figure S10: Temporal evolution of the central C=C bond of the TCNE subunit extracted from the excited state AIMD trajectories along with its Fourier Transform analyis.

Figure S11: Temporal evolution of the center of mass distance between the two monomers extracted from the excited state AIMD trajectories along with its Fourier Transform analyis.

Figure S12: Normalized distribution of the center of mass distance and the electronic S_0-S_1 energy gap.

Figure S13: Comparison of the IR spectra computed for a ground state minimum energy structure in implicit DCM solvent with and without diffuse functions for non-hydrogen atoms in the split-valence double-zeta basis set.

Figure S14: Vertical excitation energies for the first two excited electronic states calculated on conformers regularly extracted from the ground state AIMD trajectory.

Figure S15: Net charges Natural Bond Orders population analysis performed on conformers regularly extracted from the ground state AIMD trajectory.

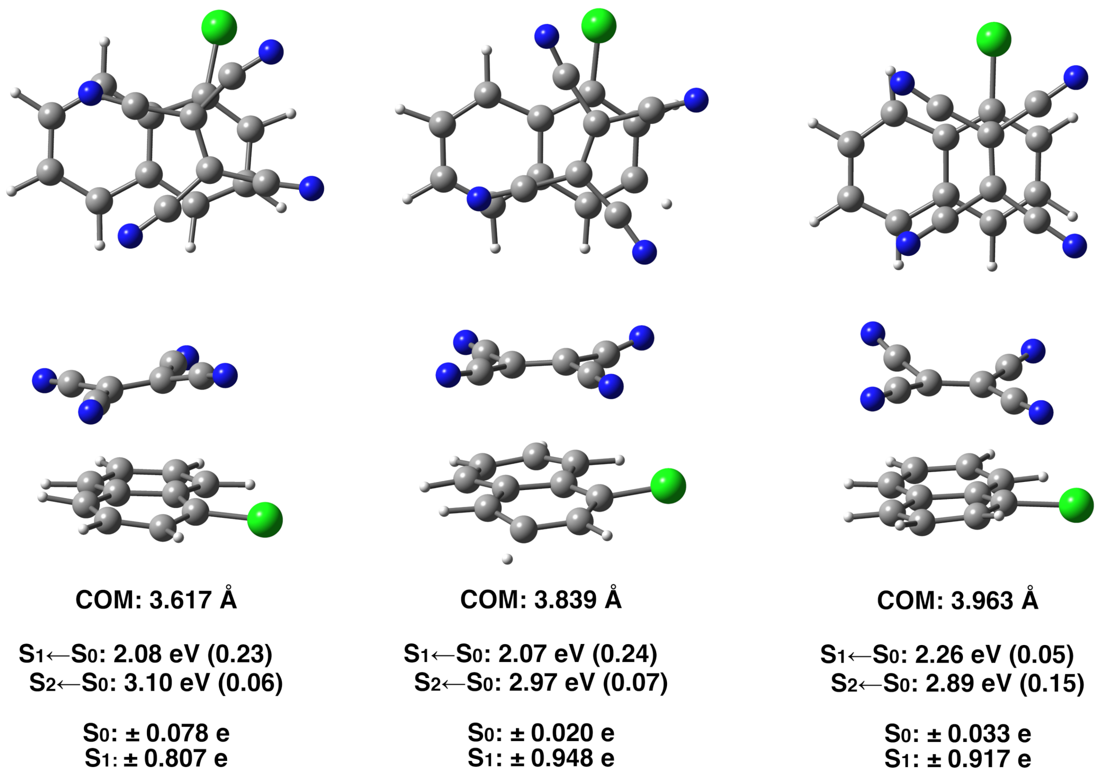

Figure S1. Initial geometries to run excited state BOMD simulations, extracted from ground state AIMD simulations for which the center of mass distances between the two monomers, TD-DFT vertical excitation energies (absolute errors with respect to experimental values are given in parenthesis) and the total NBO charge (e) for each subunit in the ground (S_0) and first singlet excited state (S_1, CT character) is given.

Table S1. Mean values of selected structural parameters, standard deviations are given in parenthesis, extracted from S_0 and S_1 *AIMD* of TCNE:1ClN CT complex. Labels are referred to Fig.1 in the main text and bond lengths are reported in Angstrom (Å).

	AIMD$_{<10ps>}$ S_0	AIMD$_{<1ps>}$ S_1
	Tetracyanoethylene	
**C=C**	1.37 (± 0.01)	1.43 (± 0.02)
**C-C**	1.43 (± 0.02)	1.41 (± 0.01)
**C≡N**	1.16 (± 0.01)	1.164 (± 0.002)
	1-Chloronaphthalene	
**C-Cl**	1.77 (± 0.04)	1.72 (± 0.02)
**C-C1**	1.43 (± 0.03)	1.42 (± 0.01)
**C-C3**	1.42 (± 0.02)	1.38 (± 0.02)
**C-C6**	1.44 (± 0.02)	1.43 (± 0.02)
**C-C9**	1.42 (± 0.03)	1.39 (± 0.02)

Time-independent Fourier spectra

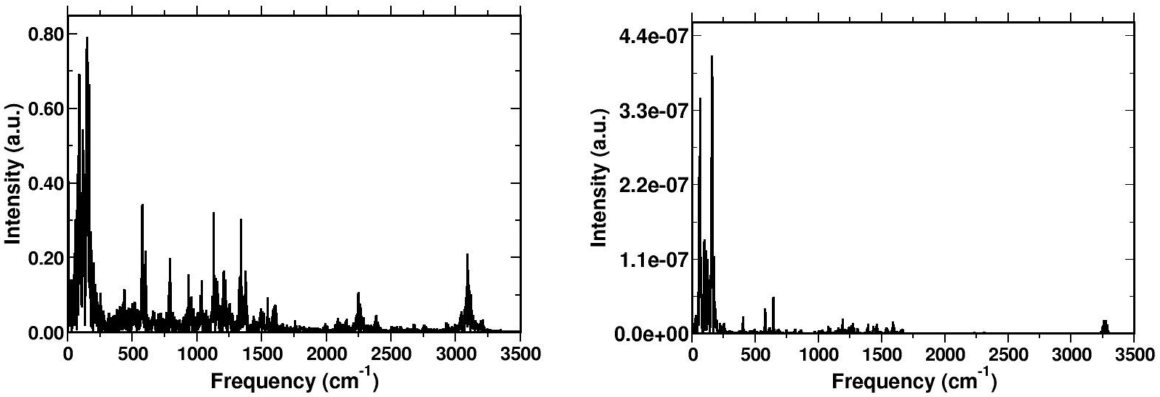

Figure S2. Time-independent spectra of the out of plane bending mode extracted from ground (left) and excited state (right) trajectory.

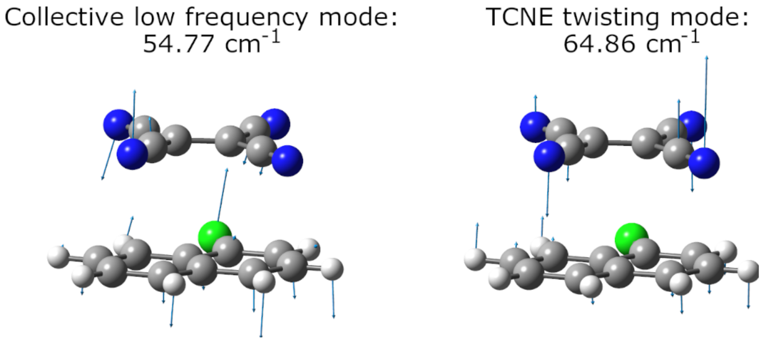

Figure S3. Low frequencies vibrational normal mode displacements observed in the excited state time-resolved Wavelet spectrum of the out of plane bending mode in Fig. 2, top right.

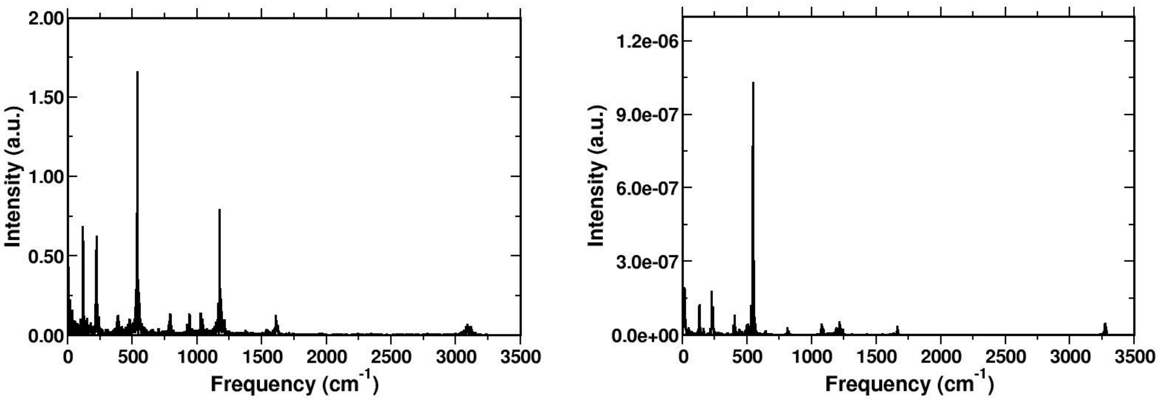

Figure S4. Time-independent spectra of the in plane bending mode extracted from ground (left) and excited state (right) trajectory.

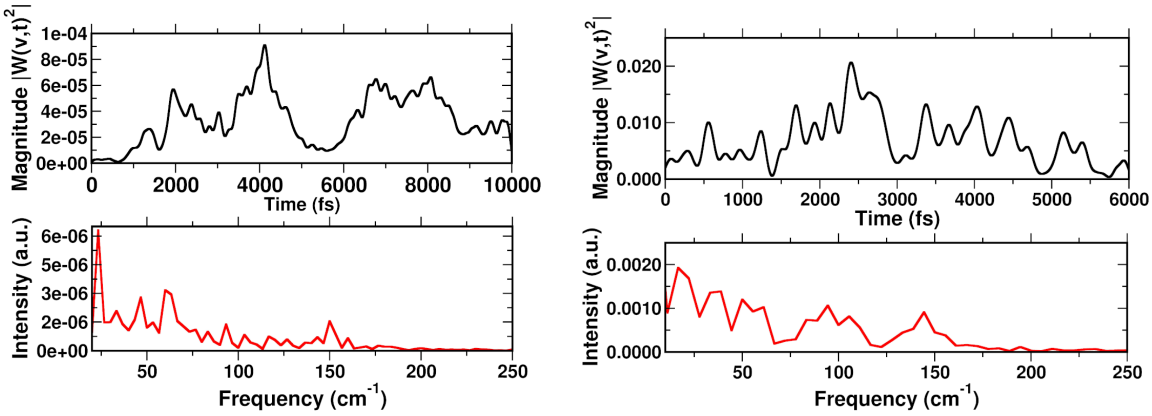

Figure S5. Magnitude fluctuations for the symmetric in plane CCN bending mode, from GS (left panel) and ES (right panel) Wavelet power spectra extracted in the time (top) and frequency domains (bottom).

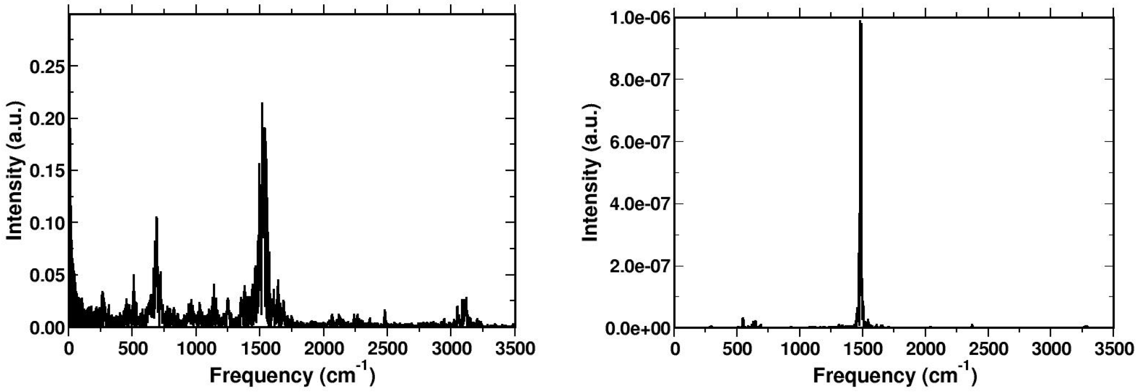

Figure S6. Time-independent spectra of the central C=C stretching mode extracted from ground (left) and excited state (right) trajectory.

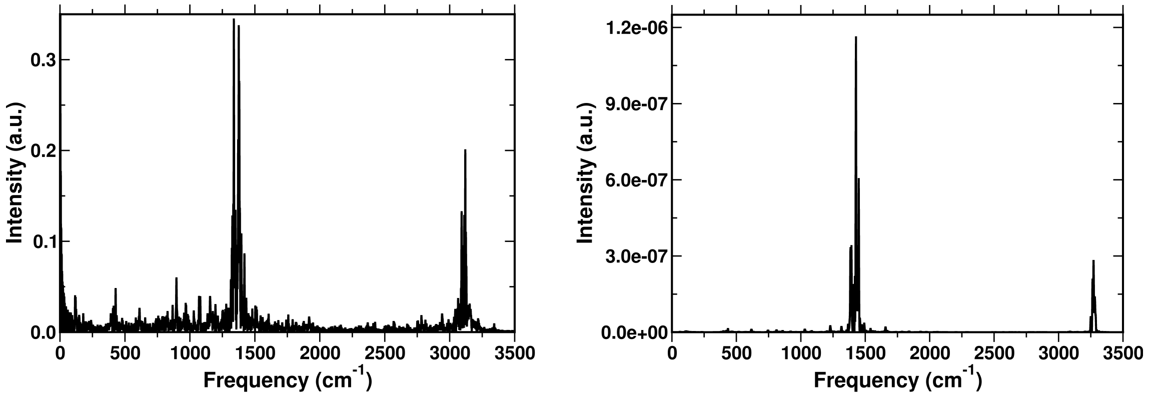

Figure S7. Time-independent Fourier spectra related to vibrational modes involving mainly the CC1 bond stretching having silenced the TCNE molecule extracted from ground (left) and excited state dynamics (right) peaked at 1338, 1380 cm^{-1} and 1392, 1428 cm^{-1}, respectively.

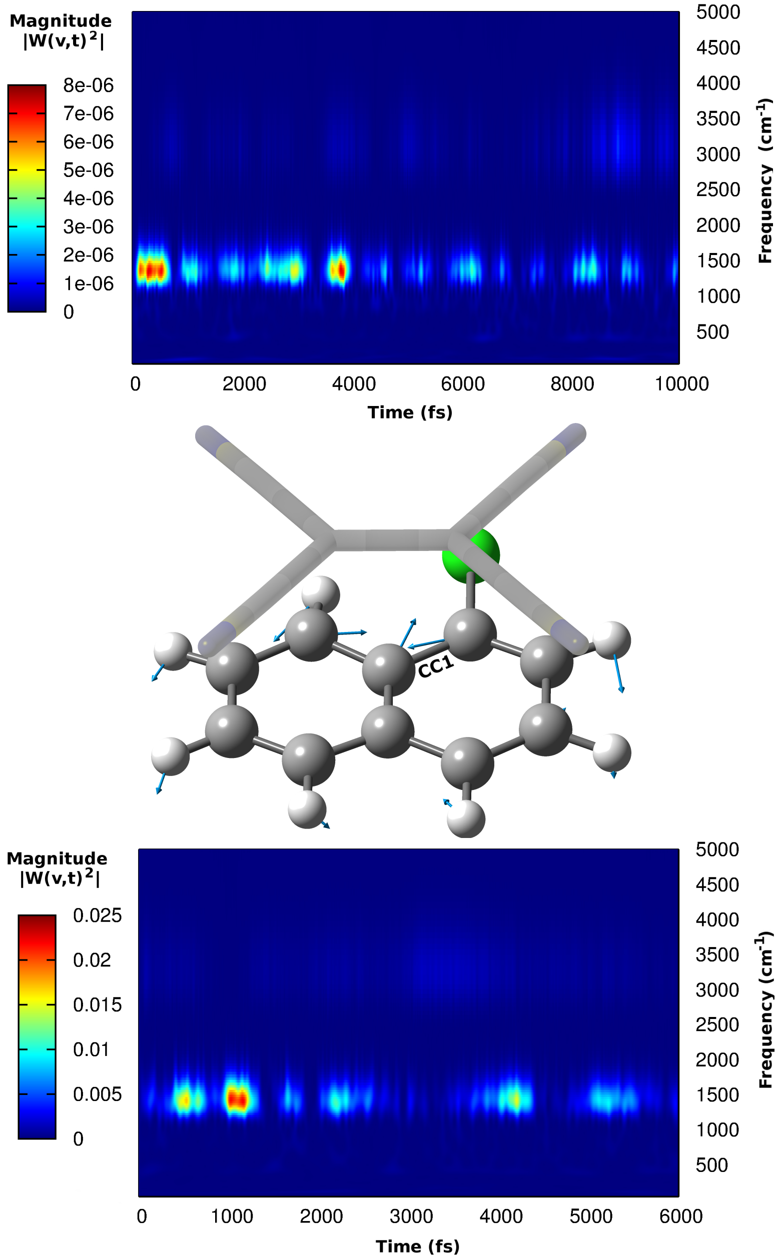

Figure S8. Wavelet spectra related to vibrational modes involving mainly the CC1 bond stretching (central panel) having silenced the TCNE molecule, reported in gray, extracted from ground (left) and excited state dynamics (right). To obtain a better visualization of the CC1 vibrational mode, for each step of the trajectory for the 1ClN coordinates and momenta rotational modes of the subsystem have been projected out by a minimization procedure of the angular momentum with respect the orientation assumed by the molecule in the first time step.

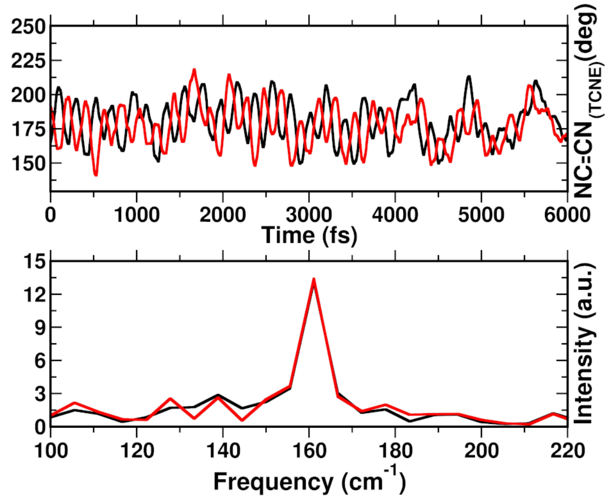

Figure S9. Temporal evolution of the NC=CN dihedral angles from excited state AIMD trajectories (top) and its Fourier transform spectrum (bottom). Average value: 180.6° and 177.3° for black and red trace, respectively.

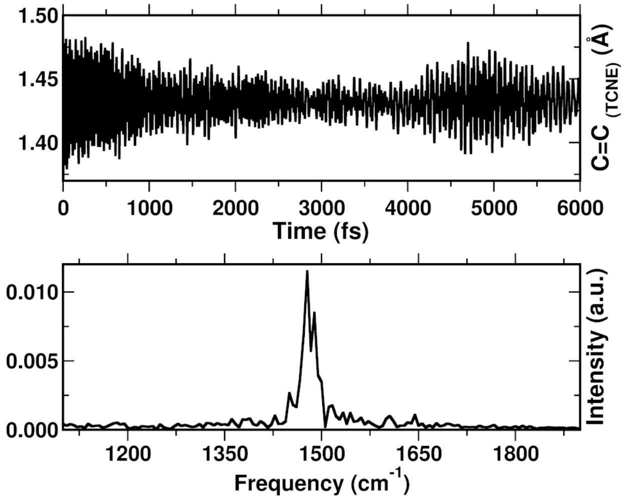

Figure S10. Temporal evolution of the C=C bond length averaged on three excited state AIMD trajectories (top) and its Fourier transform spectrum (bottom). Average value 1.43 Å.

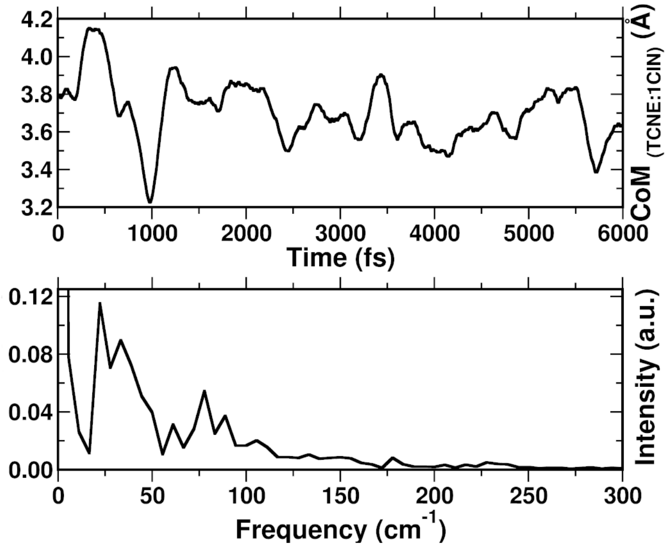

Figure S11. TCNE:1ClN center of mass distances computed averaging over the three 6ps long excited state trajectories (top) and its Fourier spectrum (bottom). Average value: 3.66 Å.

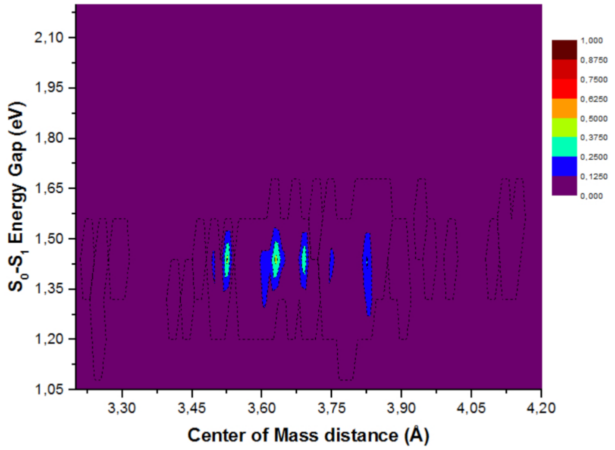

Figure S12. Normalized distribution of the center of mass distance between the TCNE:1ClN CT complex (x-axis) and the energy gap (y-axis) values between ground and first singlet excited state.

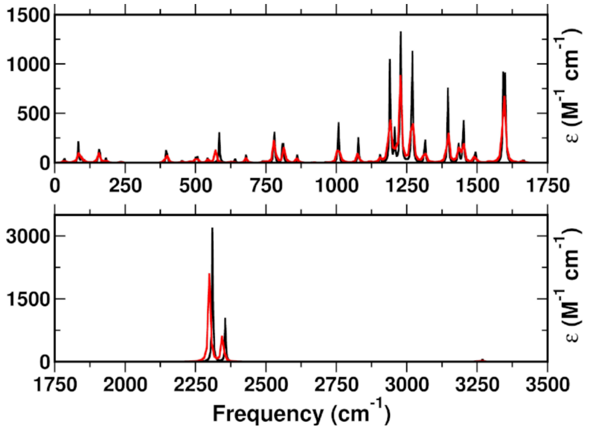

Figure S13. Harmonic vibrational frequencies (in wavenumbers, cm^{-1}) computed for a minimum energy structure at TD-CAM-B3LYP/6-31+G(d,p) (black trace) compared with TD-CAM-B3LYP/6-31G(d,p) (red trace) theory level in implicit DCM solvent. The maximum difference observed is $\approx$ $10cm^{-1}$ in the stretching region of the CN groups, 2300-2350 cm^{-1}

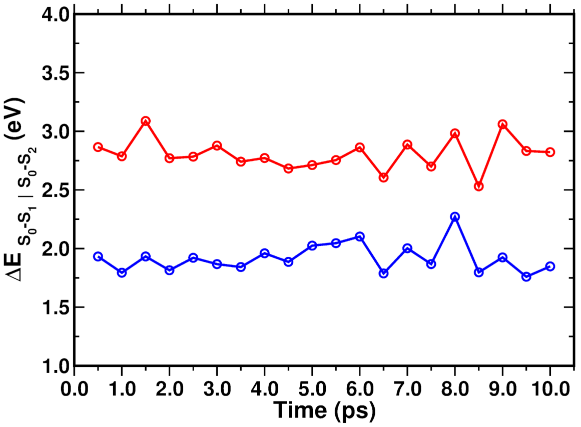

Figure S14. Vertical Excitation Energies $S_1 \leftarrow S_0$ (solid line and blue circles) and $S_2 \leftarrow S_0$ (solid line and red circles) computed on conformers extracted each 500 fs from ground state *AIMD*.

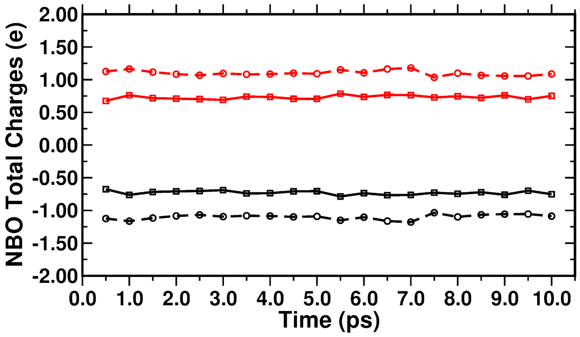

Figure S15. Ground (solid) and first singlet excited state (dashed) net charges (e) according to Natural Bond Orbitals analysis performed on twenty configurations regularly extracted every 500 fs from the ground state trajectory for the TCNE (black traces) and 1ClN (red traces) monomers.
